# Biofilm Forming *Lactobacillus*: New Challenges for the Development of Probiotics

**DOI:** 10.3390/microorganisms4030035

**Published:** 2016-09-20

**Authors:** María José Salas-Jara, Alejandra Ilabaca, Marco Vega, Apolinaria García

**Affiliations:** Laboratorio de Patogenicidad Bacteriana, Departamento de Microbiología, Facultad de Ciencias Biológicas, Universidad de Concepción, Concepción 4030000, Chile; mariajosesalasjara@gmail.com (M.J.S.-J.); aleilabaca@udec.cl (A.I.); mco.vga@gmail.com (M.V.)

**Keywords:** probiotics, biofilm, *Lactobacillus*, encapsulation

## Abstract

Probiotics are live bacteria, generally administered in food, conferring beneficial effects to the host because they help to prevent or treat diseases, the majority of which are gastrointestinal. Numerous investigations have verified the beneficial effect of probiotic strains in biofilm form, including increased resistance to temperature, gastric pH and mechanical forces to that of their planktonic counterparts. In addition, the development of new encapsulation technologies, which have exploited the properties of biofilms in the creation of double coated capsules, has given origin to fourth generation probiotics. Up to now, reviews have focused on the detrimental effects of biofilms associated with pathogenic bacteria. Therefore, this work aims to amalgamate information describing the biofilms of *Lactobacillus* strains which are used as probiotics, particularly *L. rhamnosus*, *L. plantarum*, *L. reuteri*, and *L. fermentum*. Additionally, we have reviewed the development of probiotics using technology inspired by biofilms.

## 1. Introduction

Probiotic bacteria are defined as “live microorganisms which, when administered to a host in adequate amounts, confer them health benefits” [1]. Lately, they have been the target of studies due to their potential as an alternative therapy for diseases caused by pathogenic bacteria [2]. Additionally, beneficial is their ability to form biofilms, a characteristic enabling them to resist environmental conditions, leading to the successful colonization and maintenance of their population [3]. Usually, probiotics form complex communities, known as biofilms, which possess several characteristics beneficial for the development of a microbial population facing different abiotic or biotic factors [4]. It must be taken into consideration that colonization requires the preferential adhesion of bacteria to a specific epithelium, such as the intestinal mucosa, prolonging and stabilizing their residence in the epithelium and helping to exclude pathogenic bacteria by competitive inhibition or steric impediment and having the possibility of triggering the immune response of the host cell [5]. A principle characteristic of biofilms is the formation of an extracellular polysaccharide matrix, which helps to provide protection against antibiotics and enzymes [6] and supports the generation of a microenvironment for the metabolic interaction of the population. Communication among bacteria mediated by quorum sensing, a process which may regulate gene expression, also exists [7]. A mature biofilm has greater antibacterial activity and tolerance to gastric pH than a newly formed biofilm [8]. The capacity of different bacterial species to form biofilms has been studied and observations indicate that they use diverse abiotic surfaces to support them (mainly polystyrene or glass). Furthermore, their ability to inhibit some pathogenic bacteria has been reported.

Since microorganisms introduced to a host should be protected by a physical barrier to avoid their exposure to adverse environmental conditions, another area of interest in probiotic research is bacterial encapsulation. For example, in the hypothetical case that probiotics are to be incorporated into some foods, they must be stable through time and not react with the components of the food or, if destined to the intestine, they must resist the pH of the stomach. Encapsulation techniques allow coating small quantities of a certain probiotic strain with a protecting material, usually polymeric. Encapsulated bacteria are protected against factors such as heat and humidity, maintaining their stability and viability in adverse environments, such as freezing or gastric pH [9]. During the last years, efforts have been devoted to innovate in the process of encapsulation, aiming to surpass the current limitations with planktonic bacteria to begin a fourth generation of probiotics using bacterial biofilms with a double coating and conferring beneficial features to the capsules. This could include protection against the pH of the stomach or to lyophilization [10]. This review outlines the basic concept of probiotic use as an alternative therapy to treat gastrointestinal diseases; subsequently focusing on the capacity of probiotics to form biofilms and on the advancements of encapsulation techniques of the fourth generation (biofilms), a concept that has arisen only a couple of years ago.

## 2. Probiotics

### 2.1. Probiotics, Basic Concepts

Probiotic bacteria were discovered in dairy products, their beneficial effect on the intestinal microbiota being observed by Bulgarian farmers who lived longer as a consequence of the constant and regular consumption of yoghurt [11]. Nevertheless, this relationship between yoghurt consumption and longevity has not been scientifically proven up to now. The concept of functional food was promoted by Japanese scientists studying the relationship among nutrition, sensory satisfaction, fortification and modulation of physiological systems [12]. The most widely accepted definition of probiotics is “live microorganisms which, when administered to a host in adequate amounts, confer them health benefits” [1]. In general, they are used as food supplements together with other treatments, such as antibiotics. The accepted quantity of the product to be consumed daily is in the range of 10^6^–10^7^ CFU/g [13]. Among the characteristics that microorganisms to be called effective probiotics must possess it is possible to include their capacity to increase the number of beneficial microorganisms in the gastrointestinal tract and, at the same time, to decrease the impact of pathogenic microorganisms present without causing alterations that will trigger pathology. Thus, they must be completely innocuous. In addition, there is a general strategy to select a probiotic strain, recommending that it comes from the same species of the host [14,15], followed by functional tests of the probiotic strain in order to determine if it is effective against pathogenic bacteria. Parallel assays, such as antibiotic resistance, release of toxic molecules and hemolytic potential, among others, allow its safety to be determined. After proving that potential probiotic strains are safe and that they possess antimicrobial activity, assays in cellular and animal models are performed. After all the previous experiments have demonstrated that a strain is active against the pathogen under study and that it is 100% safe, human assays can be performed. Finally, when all requested requirements are fulfilled, it is possible to start marketing the probiotic product [16]. 

Strains most widely used as probiotics belong to genera *Lactobacillus*, Streptococcus, Bifidobacterium and yeasts [17], in particular Lactic Acid Bacteria (LAB), including the genus *Lactobacillus*, are the most widely used. *Lactobacillus* are Gram positive bacteria which usually live in anaerobic environments but able to resist aerobic conditions [18]. Among the principal mechanisms of probiotic action, it is possible to find the inhibition of enteric pathogens by the production of lactic acid, hydrogen peroxide and bacteriocins; competitive exclusion of enteric pathogens by blocking adhesion sites, competition for nutrients and modulation of the immune system, including inflammation reduction [19]. They also provide benefits to the host, such as lactose intolerance alleviation; cholesterol decrease by assimilation, sustenance of the intestinal normal microbiota and dysbiosis ameliorating; suppression of toxin production, degradation of toxin receptors in the intestine, preservation of normal intestinal pH, increase intestinal motility and help to maintain the integrity of the intestine permeability [20]. In brief, probiotics are microorganisms capable of conferring benefits to the host. It must be emphasized that there is still plenty to investigate about these bacterial strains, for example their mechanisms of action, and that it is field in which Microbiology has much to explore.

### 2.2. Probiotics as an Alternative Therapy

Presently, probiotics constitute a feasible solution as an alternative therapy focused mainly in the prevention of gastrointestinal infections, but this will depend on the probiotic strain. One example is the genus *Bifidobacterium*, used to prevent gastrointestinal infections and regularly incorporated into fermented dairy products or food supplements. It has been observed to exert anti-Helicobacter pylori activity in in vitro assays, inhibiting, by competence, the adhesion of the pathogen to the mucosa [21]. Some probiotic bacteria, such as *Lactobacillus acidophilus*, *Lactobacillus casei* Shirota, *Bacillus subtilis* and *Weissella confusa*, among others, also have an antagonistic effect against *H. pylori*. Based on in vitro assays, other works have hypothesized, that probiotics stimulate the immune system through the responses of host intestinal epithelial cells [2]. The main results of studies using probiotics to treat diseases caused by pathogenic bacteria, specifically *H. pylori*, were prophylactic, reducing bacterial colonization and alleviating the gastric inflammation associated to pathogenic bacteria [22]. Additionally, recent clinical assays have concluded that there is, in fact, an improvement of *H. pylori* caused gastritis and a reduction of bacterial colonization associated with the administration of probiotics; thus, they were useful in reducing the adverse effects of antibiotics [23]. Another example is the treatment against infections caused by *Salmonella*, an important food transmitted pathogen. In vitro and in vivo studies showed the effectiveness of probiotic administration to prevent or treat *Salmonella* caused infections, ranging from mild disease to the more severe, such as enteric gastroenteritis, typhoid fever, bacteremia and septicemia [24]. In patients with typhoid fever, diarrhea can be severe enough to require hospitalization. The literature encompasses a large body of clinical evidence that describes the benefits of probiotics in helping prevent and/or treat diarrhea causing diseases [25]. For example, probiotics have been widely used to treat enterotoxigenic *Escherichia coli* (ETEC) caused intestinal infections, producing diarrhea mainly in travelers [26,27]. Among the mechanisms that explain this inhibition we can mention the competitive exclusion of ETEC because *Lactobacillus plantarum* recognizes the same adherence sites on the intestinal epithelium surface [27]. A mannose-specific adhesion has been identified in *L. plantarum* 299 (5 DSM 6595) and 299v (5 DSM 9843) strains of human intestinal origin, this adhesin could be involved in the ability to colonize the intestine. *E. coli* expresses type I fimbriae, which are involved in the specific adhesion to mannose present in epithelial cells [28,29].

Clinical trials and reference lists of hospitalized children from 1 month to 18 years old were searched in June 2013, looking for randomized controlled trials comparing the effects of the administration of probiotics, placebo or no intervention [30]. When comparing the administration of *L. rhamnosus* GG (LGG) with a placebo, reduced risks of healthcare-associated diarrhea and rotavirus gastroenteritis were observed [30].

The proposed mechanisms by which probiotics can exert their protecting or therapeutic effect against enteric pathogens include stabilization of the intestinal mucosa, increased mucous secretion and improved intestinal motility. Therefore, they interfere with the capacity to colonize and infect the mucosa, competing for nutrients and also secreting low molecular weight antimicrobial substances (bacteriocins) [31,32]. In addition, they can modulate the inflammatory response, regulating the increase of IgA and of the microbicidal activity of macrophages [33]. 

Another case is *Clostridium difficile*, in which, due to the high morbidity, mortality and increasing resistance to antibiotics, non-traditional therapies using probiotics are ideal alternatives to presently used treatments. Non-traditional therapies also constitute a preventive course of action. Nevertheless, experts sustain that it is necessary to pursue efforts to determine specific probiotic doses and formulations by high quality clinical studies in order to elucidate clear recommendations for probiotic applications that will favor health [34]. Probiotics can also act outside the gastrointestinal system. A study done by Madden-Fuentes et al. (2015) demonstrated that the combination of fluoroquinolone and probiotics used in children diminished the recurrence of urinary tract infections caused by pathogenic bacteria [35]. In general, the direct role of probiotics as an alternative or complement to antibiotic therapy, mainly in the treatment of infections of the gastrointestinal system or of other kinds, is becoming better documented as time passes. Moreover, probiotics have the potential to avoid the use of antimicrobials or to reduce the secondary effects concomitant to their use, such as antibiotic resistance or the negative effects on the health of the patients.

## 3. Biofilms of *Lactobacillus*

### 3.1. Biofilms, Basic Concepts

Bacteria can be found in nature under two forms: either freely floating planktonic bacteria or as sessile colonies of microorganisms forming biofilms. The first description of a bacterial biofilm [36] marked the beginning of a growing importance of these microbial communities [37].

A biofilm is defined by Donlan and Costerton (2002) as “a microbially derived sessile community characterized by cells that are irreversibly attached to a substratum or interface or to each other, are embedded in a matrix of extracellular polymeric substances that they have produced, and exhibit an altered phenotype with respect to growth rate and gene transcription” [38].

The formation and development of a biofilm is affected by multiple factors, including the bacterial strain, the properties of the surface and environmental parameters such as pH, nutrient concentration and temperature [39].

Five stages have been proposed during the formation of a biofilm. (i) Initial attachment; (ii) irreversible attachment; (iii) early development of biofilm architecture (microcolony formation); (iv) maturation; and (v) dispersion. In the first stage, the cell’s adhesion, strongly depends on the physiochemical properties of the bacterial cell surface. At this point, the adhesion is reversible since the attached microorganisms are not yet committed to the differentiation process which requires a series of morphological changes leading to biofilm formation [40]. Many cells can detach from the surface and return to the planktonic lifestyle [40]. The adhesion to the surface is also dependent on physicochemical properties of the surface such as texture (rough or smooth), hydrophobicity, pH and temperature [38]. In the second stage, the change from reversible to irreversible attachment involves a shift from a weak interaction of bacteria with the surface to a permanent binding with the presence of exopolysaccharide (EPS) [41]. The third stage is characterized by the aggregation of cells into microcolonies resulting from concurrent accumulation and growth of microorganisms and it is the principle stage at which EPS is produced [42]. EPS helps to strengthen the bond between bacteria and the substratum and stabilizes the colony from any environmental stress [42]. When bacteria form microcolonies they can benefit from interspecies substrate exchange and/or mutual end-product removal [43]. In the fourth stage, the biofilm matures and it develops into an organized structure which can be flat or mushroom-shaped, depending on the source of nutrients. Periods of 10 days or more are required [41]. Finally, during the fifth and last stage, cells of the biofilm will detach from the colony and transitorily return to a planktonic form, spreading out [40]. Detachment seems to be an active process allowing the colonization of new niches [44].

Microorganisms included in the biofilm show a different behavior to planktonic ones. They are more resistant to conventional treatments with antibiotics and are able to more easily evade the immune system of the host. It must be emphasized that routinely used antibiotics have been selected by their activity against planktonic bacteria. Antibiograms are designed to evaluate the sensitivity of planktonic bacteria and these results might not be extrapolated to the same bacteria when forming a biofilm. Properties making bacteria in a biofilm resistant to antibiotics and to the immune system also make them difficult or impossible to be cultured in vitro [45].

Biofilm formation by probiotic bacteria, such as *Lactobacillus* spp., is considered a beneficial property because it could promote colonization and longer permanence in the mucosa of the host, avoiding colonization by pathogenic bacteria [40]. The capacity of *Lactobacillus* to form biofilms on abiotic surfaces (glass or polystyrene) has been studied during the last years, and the results indicate that only some strains have this property [40,46,47,48,49,50,51,52,53]. It has also been demonstrated that the EPS produced by some biofilm forming strains is able to inhibit the formation of biofilms by certain pathogenic bacteria [54,55,56].

### 3.2. Biofilms and Lactobacillus rhamnosus

*Lactobacillus rhamnosus* GG (ATCC 53103) is one of the best clinically studied probiotic microorganisms. This strain is capable of adhering to the human intestinal mucosa and persisting there for more than one week after being orally ingested by healthy adults [57]. It is also able to form biofilms in vitro on an abiotic surface (polystyrene), a characteristic strongly influenced by the culture medium used and conditions associated with the gastrointestinal environment, such as low pH, high osmolarity or the presence of bile or mucin. It is able, for example, to form biofilms when cultured in Man Rogosa Sharpe (MRS) medium without glucose (main source of Carbon for LAB) while the presence of mucin increases its capacity to form a biofilm by 20%. In contrast, a low pH decreases its capacity to form a biofilm [46]. Other *L. rhamnosus* strains isolated from dairy products, such as *L. rhamnosus* 183, have also shown a strong capacity to form biofilms in MRS medium on polystyrene surfaces and, as biofilms, they are able to strongly inhibit pathogenic bacteria such as *Escherichia coli* and *Salmonella* spp. [47]. Lebeer et al. (2009), studied the specific role of EPS molecules in the adherence to mucus, epithelial cells and biofilm formation by *L. rhamnosus* and demonstrated that a mutant obtained for the *welE* gene, related to EPS biosynthesis, increased biofilm formation when compared to the wild type strain [58]. Although the reason for these results is unknown, it is argued that the absence of EPS might expose adhesins favoring the binding to several surfaces. The role of lipoteichoic acid in biofilm formation was also analyzed, but studies by Velez et al. (2007) established that the composition of this acid did not affect the ability of this strain to form biofilms [59]. Leccese et al. (2016) evaluated the kinetics of biofilm formation and the chemical nature of the biofilm matrix formed by *L. rhamnosus* CRL 1332, demonstrating that protease, proteinase K and α-amylase detached this strain from the biofilm because the matrix contains large amounts of polysaccharides, carbohydrates and proteins [60]. O Since biofilm formation is related to bacterial communication by quorum sensing, the role of the *luxS* gene on growth and biofilm formation by *L. rhamnosus* GG has been analyzed in different contexts. It was demonstrated that *luxS* mutants have a lower capacity to form biofilms than the wild type strain [46,61].

### 3.3. Biofilms and Lactobacillus plantarum

Another *Lactobacillus* species widely studied for its capacity to form biofilms is *L. plantarum*. Kubota et al. (2008) studied the capacity of three LAB strains to form biofilms, including *L. plantarum subsp. plantarum* JCM1149 among them. They showed that it was able to form biofilms in MRS broth on a glass surface and that it was more resistant to acetic acid and ethanol than planktonic bacteria [48]. The change of the resistance profile to environmental stress conditions of *L. plantarum subsp. plantarum* JCM1149 forming biofilms as compared to the planktonic state was also studied [49], and it was demonstrated that resistance to organic acids, ethanol and sodium hypochlorite was greater in the biofilm status, suggesting that they pose a risk for food deterioration and therefore demonstrate the importance to control LAB biofilms in the food industry. Nevertheless, most biofilm studies are related to the intestine. One of them showed that there are proteins associated to colonization by *L. plantarum*, corroborated with evidence that biofilm formation and its propagation are regulated by several proteins [62]. The Msa protein participates in adhesion and the enzyme glyceraldehyde-3-phosphate dehydrogenase (GAPDH), associated to the cell surface of *L. plantarum* LA318, mediates the adhesion to human colon cells recognizing a sugar and acting as a lectin-type protein [63]. It has been also shown that *L. plantarum* is capable of increasing the integrity of the epithelial barrier [64]. The expression of mucin and adherence genes is dependent on the direct cellular contact between *L. plantarum* and epithelial cells of the intestine [65,66]. Furthermore, it has been found that bacteria which colonize the gastrointestinal tract generally grow well in biofilms [67]. Thus, there is a series of mechanisms to be followed when colonizing the tissue, an early stage necessary in the formation of a biofilm. After adherence, bacteria must be able to aggregate and colonize the intestine. *L. plantarum* LP9 demonstrated a 65% auto-aggregation. Cellular aggregation contributes not only to transitory colonization but also to providing a protective shield when forming a biofilm on the tissue of the host [66].

Fernández et al. (2015) studied the capacity of *L. plantarum* WCFS1 to form biofilms, showing that this strain is highly affected by the composition of the culture medium, growth temperature and time of maturation, being Brain Heart Infusion (BHI) medium supplemented with manganese and glucose the one showing the best results [53]. They also showed that biofilms were highly sensitive to treatment with proteinase K, suggesting an important role of proteins or proteinaceous material on surface colonization. Interestingly, the presence of adhesive molecules on the cellular surface of probiotics has been related to the ability to confer health benefits to the host. Vastano et al. (2015) studied the role of Eno A1 Enolase by means of a comparative analysis between *L. plantarum* LM3 (wild type) and the isogenic mutant strain LM3-CC1 (ΔenoA1). They reported that Eno A1 affects the ability of the bacterium to modulate the immune response in Caco-2 cells, inducing the secretion of pro-inflammatory (IL-6) and anti-inflammatory (IL-10 and TGF-β) cytokines and the antimicrobial peptide human β-defensin-2 (HBD-2). They also analyzed the capacity of both strains to form biofilms, showing a 65% decrease in biofilm formation by the mutant LM3-CC1 (ΔenoA1) when compared to the wild type strain [68].

Quorum sensing (QS) is mediated by mechanisms such as the *luxS* gene and the pheromone peptide plantaricin A (Plna), both of which could play a fundamental role in the regulation of the microbial interactions in intestinal human ecosystems. Calasso et al. (2013) investigated the exoproteome of *L. plantarum* DC400 when cultured in the presence of Plna or when co-cultured with other *Lactobacillus* species, demonstrating that *L. sanfranciscensis* DPPMA174 increased notably the capacity of *L. plantarum* DC400 to adhere to Caco-2 cells and to form biofilms. Furthermore, the interaction between these two strains allowed *L. plantarum* DC400 to increase the levels of proteins responsible for stress resistance, adherence an immunomodulation (for example, GroEL and/or DnaK) [69]. De Angelis et al. (2015) studied the exoproteome of *L. plantarum* DB200, selecting this strain for its capacity to form biofilms and to adhere to Caco-2 cells. The analysis of proteins by two-dimensional difference gel electrophoresis (2D-DIGE) showed a differential exoproteome between cells forming biofilm and planktonic cultures. Concordantly, the high levels of expression of stress proteins (Dnak, GroEL, ClpP, GroES and catalase) in cells forming a biofilm showed their better survival under environmental stress conditions (heat, acid and ethanol) [70].

It has also been seen that the supernatant of *L. plantarum* WCFS1 and *L. plantarum* NA7, when forming a biofilm, produce molecules inhibitory for pathogens (*E. coli* O157:H7, *Salmonella enterica* serovar Enteritidis, *Staphylococcus aureus* and *Listeria monocytogenes*). Furthermore, the supernatant of *Lactobacillus* cultured as a biofilm, but not in planktonic status, suppressed TNF-α production by LPS activated human monocytoid cells. Thus, *Lactobacillus* in biofilm status is associated to strain dependent beneficial probiotic properties [52].

### 3.4. Biofilms and Lactobacillus reuteri

The capacity of *L. reuteri* to form biofilms has been a reason to subject it to considerable studies. An investigation done by Jones et al. (2009) addressed the ability of several *L. reuteri* strains to form biofilms, as well as their immunomodulating activity [50]. Two of the strains studied (*L. reuteri* ATCC PTA 6475 and *L. reuteri* ATCC PTA 5289) showed the best absorbance values and, therefore, formed denser biofilms. Their immunomodulating capacity was studied adding the supernatant of biofilms to a culture of THP-1 human monocytoid cells stimulated by LPS, and observations showed that they were capable of modulating the production of cytokines, suppressing TNF production and also producing an antibacterial agent that they called reuterin. The anti-pathogenic activity of reuterin inhibited a wide spectrum of microorganisms, including Gram positive and Gram negative bacteria, fungi and protozoa. Shu-wei et al. (2014) studied a new two-component regulatory system codified by *bfrKRT* and *cemAKR* genes and investigated its influence in biofilm formation by *L. reuteri* 100-23. In this study, they observed that disruption of these genes affected, depending on the Carbon source, the formation of biofilms. The suppression of *bfrK* or *cemA* genes improved in vitro biofilm formation in a saccharose dependent fashion. Glucose dependent biofilm formation was particularly enhanced by *cemK* suppression [71].

Most studies on this species are related to the intestinal environment. A study demonstrated that *L. reuteri* RC-14 was able to penetrate, as a co-aggregate, mature *E. coli* biofilms, annihilating this pathogen and becoming part to the biofilm [72]. The in vivo adhesion of *L. reuteri* to mouse intestine, which is mediated by specific adhesins, has been observed. Nonetheless, other factors may contribute to the adhesion specificity and they include adaptations to environmental conditions (urease and protease clusters and biofilm forming factors) and its regulation in the detection of the quorum [73].

Proteins involved in adherence and biofilm formation on epithelia have been reported in *L. reuteri* strains from different origin, such as: Collagen-binding protein (CnBP/MapA), Mucus-binding protein (Mub), Large Surface protein (Lsp) and Glycosyl-transferases (GtfA/Inu) [74]. In *L. reuteri* 100-23, a strain from rodent origin, SRRP was identified as the only protein secreted by SecA2-SecY2 and it is essential for biofilm formation [75].

A study done by Terraf et al. (2012) showed that the capacity to form biofilms is significantly influenced by the strain, culture medium, concentration of the inoculums and chemical nature of the support used for the study. For example, scanning electron microscope (SEM) observations revealed that *L. rhamnosus* CRL 1332 and *L. reuteri* CRL 1324 formed a highly structured biofilm, but *L. reuteri* CRL 1324 was the only strain showing elevated quantity of extracellular material [40]. In addition to studying *L. rhamnosus*, Leccese et al. (2016) evaluated the kinetics of biofilm formation and the chemical nature of the biofilm matrix formed by *L. reuteri* CRL 1324. They demonstrated that protease, proteinase K, α-chymotrypsin and trypsin treatments efficiently detached this strain from the biofilm because proteins are one of the main components of the biofilm matrix of this strain [60]. Finally, Olson et al. (2016) studied a strain of *L. reuteri* grown as a biofilm on microspheres, concluding that a single dose of *L. reuteri* grown on biocompatible microspheres significantly reduced the incidence and severity of necrotizing enterocolitis (NEC) [76].

### 3.5. Biofilms and Lactobacillus fermentum

Other studies have been conducted with *L. fermentum.* There is evidence of the probiotic characteristics of this species [77,78], either inhibiting the adhesion of pathogens [79] or producing compounds such as hydrogen peroxide, bacteriocins and bio-tensioactives that inhibit the growth of intestinal or urogenital pathogens [80,81]. A necessary condition prior to colonization by *L. fermentum* is the preferential adhesion to the intestinal or oral mucosa, aiding formation of the biofilm and to exclude, by competitive inhibition, pathogenic bacteria [5].

Aoudia et al. (2016) studied the binding of biofilms of different probiotic species in order to analyze their relationships, demonstrating that *L. plantarum* and *L. fermentum* strains are able to grow as biofilms on abiotic surfaces, but the biomass density differs between both strains. The biofilm of *L. fermentum* with other *Lactobacillus* is able to potentiate probiotic capacity because they resist gastrointestinal conditions; they are immunomodulators and inhibit the growth of pathogens (*E. coli* O157:H7, *S. enterica* serovar Enteritidis, *S. aureus* and *L. monocytogenes*) [52].

## 4. Development of New Technologies (Probiotic Encapsulation)

After the benefit of probiotics was discovered, their commercialization, in planktonic form, begun in dairy products such as yogurt or milk. This corresponded to probiotic products of the first generation, a concept defined as non-coated microorganisms which may be in planktonic state and lyophilized. Later on, researchers began to detect certain problems with these products, such as the limited survival of probiotic bacteria during food storage or in the gastrointestinal tract [82]. In the search to solve this problem, the second generation of probiotic products was created, corresponding to lyophilized microorganisms in pharmaceutical capsules in which a polymeric coat or synthetic, semi-synthetic or natural pharmaceutical excipients were used [83,84]. Nevertheless, new problems, such as the low sustained and prolonged liberation of the probiotics strains in the gastrointestinal tract, arose. New technologies, both in the coating material used as well as in the methodologies required to produce probiotic foods, such as encapsulation or microencapsulation, were created to increase cell viability in dairy products and in the gastrointestinal tract [85]. Thus, the third generation of probiotic products, defined as microorganisms encapsulated or microencapsulated with only one polymeric matrix, using synthetic, semi-synthetic or natural polymers, was created [82,85].

Third generation probiotic products were accompanied by new problems, the main one being that they do not act specifically at the target where their probiotic activity is necessary. In order to solve this problem, an additional polymeric coating, in which microorganisms are inserted, was added creating the concept of fourth generation probiotic product. The low sustained and prolonged liberation in the specific target site problem was solved and furthermore, the tolerance to certain environments, such as high temperatures, low temperature storage and gastrointestinal juice, was increased [10,86,87,88].

Encapsulation is defined as the physicochemical or mechanic process to trap substances in a certain material and produce particles with diameters in the order of nanometers to millimeters [85]. The purpose of encapsulation is not only to protect probiotics from external environments but also to achieve their liberation at the target site in a metabolically active condition [85]. Some of the most used techniques to encapsulate or microencapsulate bacteria, among others, are extrusion, emulsification, spray-drying and coacervation [82,85,89,90]. The oldest of them is extrusion. In this technique, bacterial cells are mixed with a polymeric solution and then extruded, through an orifice as small drops into a gelling solution [13]. Solid capsules are obtained by contact of the polymer solution with the gelling solution, cooling or a combination of both [89,90]. The main advantages of extrusion are its simplicity, low cost and mild temperatures assuring a better viability of bacteria [82].

Among others, the polymers alginate, chitosan, carrageenan and gelatin are used to encapsulate [13,85]. Alginate is a naturally derived polysaccharide extracted from various algae species and it is composed of β-d-mannuronic and α-l-guluronic acids. Calcium alginate is the most used material to microencapsulate probiotics because it is nontoxic, biocompatible and of low cost [13]. The disadvantage of alginate is its very low resistance to the gastric environment [91]. Therefore, it is mixed with other materials, such as a double coating, obtained by ionic exchange, of locust gum and chitosan, previously demonstrated to resist well gastric acid [87]. Chitosan is a linear polysaccharide composed by glucosamine units able to polymerize in a reticulating agent in the presence of anions and polyanions [85].

It is worth mentioning that alginate capsules coated with chitosan are the most widely used for probiotics which target the gastrointestinal tract. Previous investigations showed that capsules are good to maintain the viability of bacterial cells and to resist the gastrointestinal environments; moreover, it is also known that they are good vectors to carry viable cells specifically to the colon [10,86,87,88,92]. Khalil et al. (2015) demonstrated that alginate coated microcapsules help LAB to resist stressful environments, reduce the loss of viability and maintain the activity of strains evaluated against *H. pylori* [93]. Kammani et al. (2011) encapsulated *Streptococcus phocae* P180 strain, observing an important survival of the cells and that a high activity of their bacteriocins was maintained after storage at −20 °C for six months [94]. Additionally, Cheow and Hadinoto (2013) encapsulated *L. rhamnosus* GG strain, as a biofilm, with a double coating in order to test if a better resistance to stressful environments was achieved. They concluded that the strain as biofilm and with double coating was more resistant than the double coated planktonic strain [10]. Later on, Cheow et al. (2014), tested alginate mixed with other polymers searching for a better resistance to gastric juice and concluded that the chitosan coated alginate and locust gum mixture provided the best resistance to stressful environments and also increased probiotic viability in a simulated gastric juice [87]. Kiew et al. (2014), using the same double coated strain, demonstrated that the culture conditions achieving a better development of the biofilm were reached after 5 days of culture in AOAC medium [86]. Considering that starch has prebiotic properties and it also increases the resistance of biofilms, Cheow et al. (2016) tested alginate mixed with two types of starch concluding that modified 0.6% (*w*/*v*) waxy starch coated with chitosan increased tolerance of *L. rhamnosus* GG strain, as a biofilm, to lyophilization, high temperatures and cold storage [88].

At a low pH, alginate-chitosan capsules maintain their ionic bonds to beads, remaining intact the bead matrix material. After transfer to the neutral pH of the intestine, the anionic alginate in the Ca-alginate-chitosan complex is displaced by hydroxyl ions [95] resulting in the releasing of therapeutic molecules by polymer degradation [96,97]. As a result, the complex dissociates, the matrix erodes, and the protein is released in the surrounding fluid [13]. Based on the previous report, we can postulate that in the case of bacteria, the capsule would behave in a similar manner, liberating them in the intestine.

## 5. Conclusions and Projections

Numerous studies have demonstrated that biofilm formation by LAB is a beneficial property because it promotes colonization and a longer permanence of LAB in the mucosa of the host. Biofilm formation by LAB also impedes colonization by pathogenic bacteria through different mechanisms including immunomodulation. Nevertheless, this is a strain specific and non-extrapolative property, requiring the individual study of each strain. The development of new technologies of encapsulation to maintain the viability of probiotics and to allow them to arrive in an intact form to their target site have not yet been completely successful. Considering the increased bacterial viability in stressful environments, such as high temperatures and lyophilization, recent studies have focused on biofilm forming encapsulated bacteria rather than planktonic ones. Further studies on encapsulated biofilm forming strains of the genus *Lactobacillus* are necessary in order to determine the mechanism of action of each particular biofilm forming strain against clinically important pathogens. These studies should firstly use in vitro models on abiotic surfaces and subsequently in vivo models to lead to their successful commercialization.
